# Analysis of strength failure in gangue-based cementitious backfill body from a microscopic perspective

**DOI:** 10.1371/journal.pone.0300102

**Published:** 2024-04-01

**Authors:** Hao Wang, Qi Wang, Tongwen Gao, Jun Wang, Chi Sun, Zhongmin Ji, Jian Meng, Burui Ta

**Affiliations:** 1 School of Civil Engineering, Zhengzhou University of Technology, Zhengzhou, Henan, China; 2 Henan Women’s Vocational College, Zhengzhou, Henan, China; 3 School of Civil Engineering, Henan Polytechnic University, Jiaozuo, Henan, China; 4 Northwest Geological Exploration Institute, China Metallurgical Geology Bureau, Xi’an, Shanxi, China; Jamia Millia Islamia, INDIA

## Abstract

Backfill mining is an effective way to solve environment pollute, surface subsidence, and groundwater system damage which caused by coal mining. However, the complex underground environment may change the physical and mechanical properties of the backfill body, which poses a risk of strength failure. This paper analyzed the failure of gangue-based cemented backfill body which made up of gangue and fly ash. The results show that physicochemical reactions will generate quartz, kaolinite, and other high-strength substances; hydration reaction between the fine particles will generate hydrocalcium silicate and other C-S-H gels, they wrapped gangues as a whole, which provide a high strength of the cemented backfill body. Several experiments were carried out in order to find the reason for failure in samples under loads. The conclusion drawn as following: (1) When the load is large, the cracks extend from the surface of the samples to the interior, at the same time, the length and width of the cracks increasing obviously and connecting as net. Especially the external load exceeds the peak intensity. (2) The relationship between sample failure and pores is weak, but obvious with crack development, especially the cracks connected as a net. (3) The interface structure formed by gangue is an important source of crack development and, thus, will stimulate the development of cracks.

## Introduction

Mining meet energy needs and will cause surface subsidence and destroy underground water systems for the overlying strata moving of goaf areas [1, 2]. Furthermore, coal gangue wastes a large number of land resources and pollutes the environment [3, 4]. Backfill mining can effectively control the movement of rock layers and surface subsidence while also solving the environmental pollution problem caused by the discharge of gangue [5, 6]. The role to keep rock layers stable come from the compressive strength of backfill body. When it is damaged, the rock layers will fracture and the surface will subsidence.

There are pores and cracks in the interiors of materials such as backfill body and rock masses. The characteristics and spatial distribution of the pores and cracks will affect strength, permeability, and durability [7, 8]. Zhou [9] found that the geometric parameters of the original cracks and die angles of rock samples have a significant impact on their peak force by using a digital image correlation analysis (DIC) system and compression shear tests. At the same time, these micropores and cracks can develop under the influence of external factors. As the research shows, the physical properties of concrete are significantly influenced by their component proportions. The phenomenon can be extended to similar materials [10]. Liu found that the proportion of impermeable pores in a filling material increases with an increase in the proportion of bound sand, resulting in denser hydration products and improved mechanical properties [11].

Unlike with coal and rock, backfill body contains many interface structures and exhibit non-uniformity in density because of the gangue inside. In addition, backfill body was deeply buried underground and subject to multiple field coupling effects, which may result in strength damage or failure [11, 12]. Zhou [13] found that the proportion and size of sand both have a significant impact on the crack processes and failure modes of concrete. When multiple fields and confining pressure are coupled, changes in the micropores and cracks of coal will affect its permeability [14]. Li [15] found that using modified cement grouting can improve the microstructure and bonding strength of the interface between slurry and coal, thereby enhancing the stability of crushed coal at the macrolevel. Liu [16] found that the failure of gangue paste mainly occurs at the interface between coarse aggregate and cementitious materials.

Fu [17] and Li [18] believe that the failure of backfill materials involves four stages: microcrack closure stage, linear elastic stage, microcrack propagation stage, and crack penetration failure stage. Guo [19] found that the failure mode of gangue-cemented backfill gradually transforms from splitting failure to shear failure as the size increases. Zhang [20] found that the permeability of gangue-cemented backfill increases with an increase in the time spent in an acid environment and osmotic pressure and decreases with an increase in axial pressure. The cemented backfill material has obvious elastic and brittle characteristics [21]; as the samples damaged, cracks develop and connect, perhaps forming a network.

Computer tomography (CT) technology can achieve non-destructive and non-invasive research on the structural characteristics and spatial distribution of concrete components. Chen [22–24] presented the quantitative distribution of full-scale pores inside fine sandstone by CT technology, and their conclusion can be used to evaluate its permeability and reservoir quality. Yu [25] clarified the inner relationship between deformation and structural characteristics using X-ray CT scanning, MIP (mercury intrusion porosimetry) and BET (low temperature nitrogen adsorption). Wei [26] and Li [27] used CT technology to quantitatively analyze the pores and fractures in shale and lamellar shale. Wei [28, 29] complemented scanning uniaxially compressed coal samples with *μ*-CT and obtained 3D visualization of the crack network model. Furthermore, CT could visually detect the distribution of aggregates, mortar, and pores in concrete [30]. Wong [31] studied the evolution of internal pores and aggregate states in ordinary and high-strength concrete cylindrical specimens under different loading states. Liu [32] scanned concrete specimens under uniaxial compression using industrial CT and obtained 3D visualization models of concrete pores and cracks at different loading stages. Fu [33] obtained a 3D model of a coal rock mineral structure via CT scanning and iterative reconstruction; that provides conditions for study the influence of the geometric morphology, size, and distribution of mineral structures in space on the macroscopic mechanical behavior and deformation and failure characteristics of coal and rock. Liu [34] found that the freeze-thaw effect increased the porosity of the soil 3D microstructure, especially the pores with greater effective diameters; and the pore-throat networks became complex because of more branch paths and throats happens. Wildenschild [35] obtained 3D model within individual pores of subsurface porous media joint X-ray microtomographic imaging and multiphase flow.

The physical of the backfill body are different for different filling materials. For example, some of the backfill body have large aggregate particles. And the strength, porosity and permeability of the backfill bod have been studied from a microscopic point of view using techniques such as SEM and XRD [36–38]. The above literatures found a positive correlation between gangue particles and backfill body failure from a macroscopic perspective based on experiments. Some literatures revealed the C-S-H structure is an important source of the backfill body strength from a microscopic perspective. The above research shows that the particle size of gangue has a significant impact on the strength of cemented backfill body. This paper attempts to explain the phenomenon from a microscopic perspective. The research displayed the internal microstructure of the backfill body under different loads by CT and SEM techniques, and discovered a series of interesting phenomena. Gangue forms interface structure in the cemented backfill body, and its size is significantly related to the particle size of the gangue. The interface structure plays a significant role of the backfill body damage. The research demonstrates the particle size of gangue affects the strength of cemented backfill body from a microscopic perspective which can be used to evaluate the environmental effects of coalmine gangue-cemented backfill.

## Experiments

### Background

The gangue-based cemented backfill slurry made up of coarse aggregate (broken gangue, max particle size less than 1~2*cm*), fine aggregate (fly ash), and cementitious material (cement). The dense slurry (concentration of 72%~78%) transported to the goaf by pipelines, and forming high compressive strength to control the movement of rock layers quickly [39].

### Material composition analysis

The experimental equipment included industrial computerized tomography (YXLON FF85 CT, Hamburg, Germany) and a pressure servo (YongCe, Jinan, China). The experiments were conducted at room temperature. Tables 1 and 2 [41] are the chemical composition and content of the coal gangue and fly ash samples which analyzed by X-ray and ICP-OES (inductively coupled plasma optical emission spectrometer).

**Table 1:** Chemical components of fly ash.

Chemical Components	SiO_2_	Fe_2_O_3_	TiO_2_	Al_2_O_3_	CaO	MgO	P_2_O_5_	K_2_O	LOI
**Contents**	46.50	4.56	1.55	30.58	7.89	0.76	0.21	0.96	6.99

**Table 2:** Chemical components of coal gangue.

Chemical Components	SiO_2_	Fe_2_O_3_	PbO	Al_2_O_3_	CaO	MgO	P_2_O_5_	K_2_O	S	LOI
**Contents**	49.22	6.21	0.73	22.96	4.88	0.78	0.12	1.2	0.8	13.1

### Strength tests

#### Results of strength tests

In order to highlight the influence of interface structure formed by gangue on the strength of the backfill body, the gangue particles were filtered into 5 groups which represented as A (<0.1 cm), B (0.1 cm~0.5 cm), C (0.5 cm~1.0 cm), D (1.0 cm~1.5 cm), and E (1.5 cm~2.0 cm). Based on previous research [40, 41], the slurry concentration is 78%, and the mass ratio of coarse aggregate: fine aggregate: cementitious material (cement) is 8:3:1. Make three samples for each group of particles and take the average strength value to increase accuracy.

**Fig 1:**
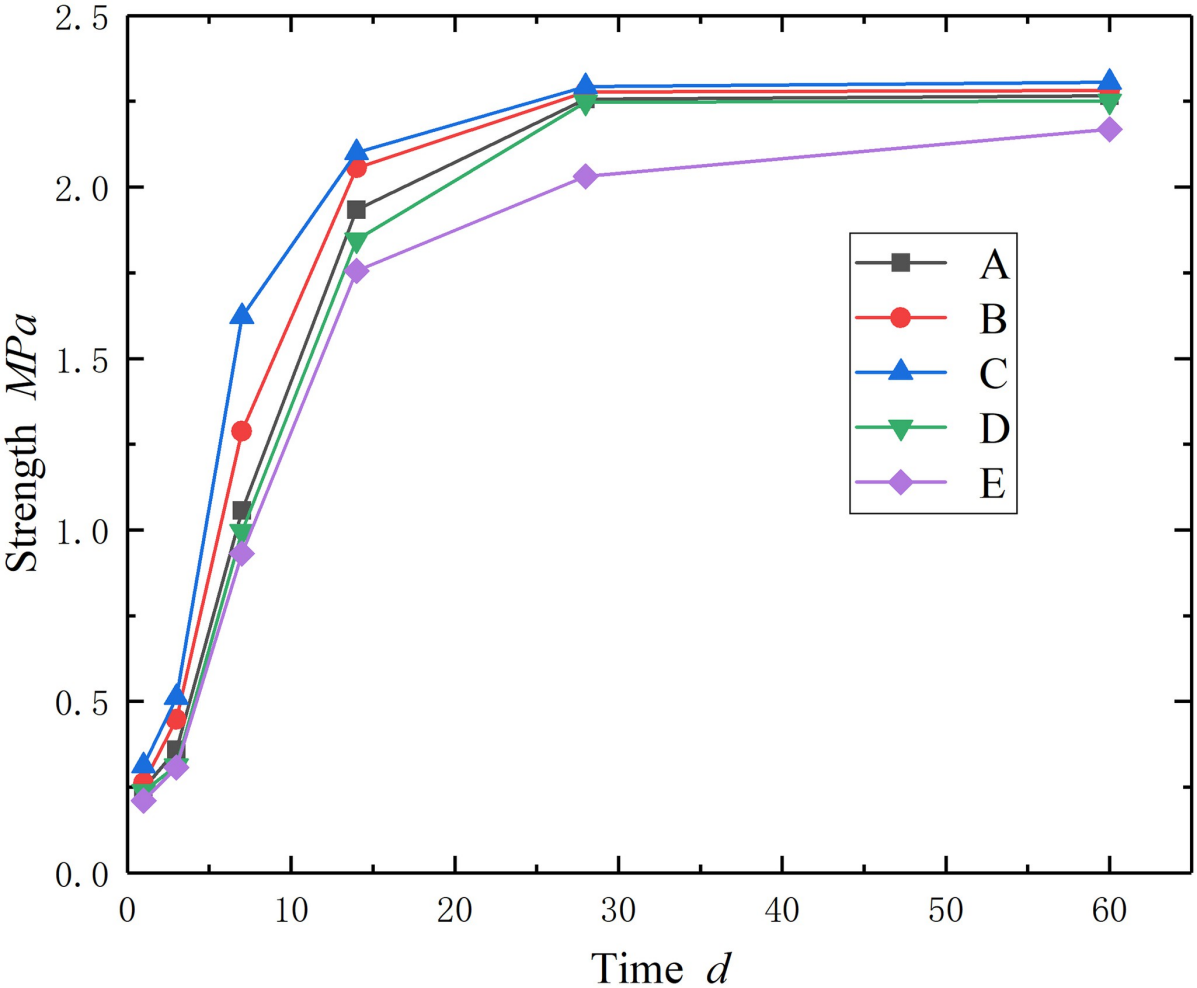
Strength versus particle sizes.

The curves of compressive strength versus curing time of the samples with different particle sizes are shown in Fig 1.

The following phenomenon can be observed: (1) The strength of the backfill body increased quickly at the first 14 days and almost reached peak strength at day 28. And then appears in a gently increasing trend. All samples exhibited similar phenomena, no matter the particle size. (2) Table 3 shows the strength versus particle size at day 28. Taking group C particle size as the boundary point, the strength of small particle size sample keeps an approximately stable trend with the particle size increase, the strength of large particle size samples decreases with the particle size increase. The minimum strength is approximately 10% lower than the maximum strength.

**Table 3:** Strength versus particle size.

Group	A	B	C	D	E
Strength (*Mpa*)	2.256	2.278	2.293	2.248	2.031

#### Analysis of strength sources

**Fig 2:**
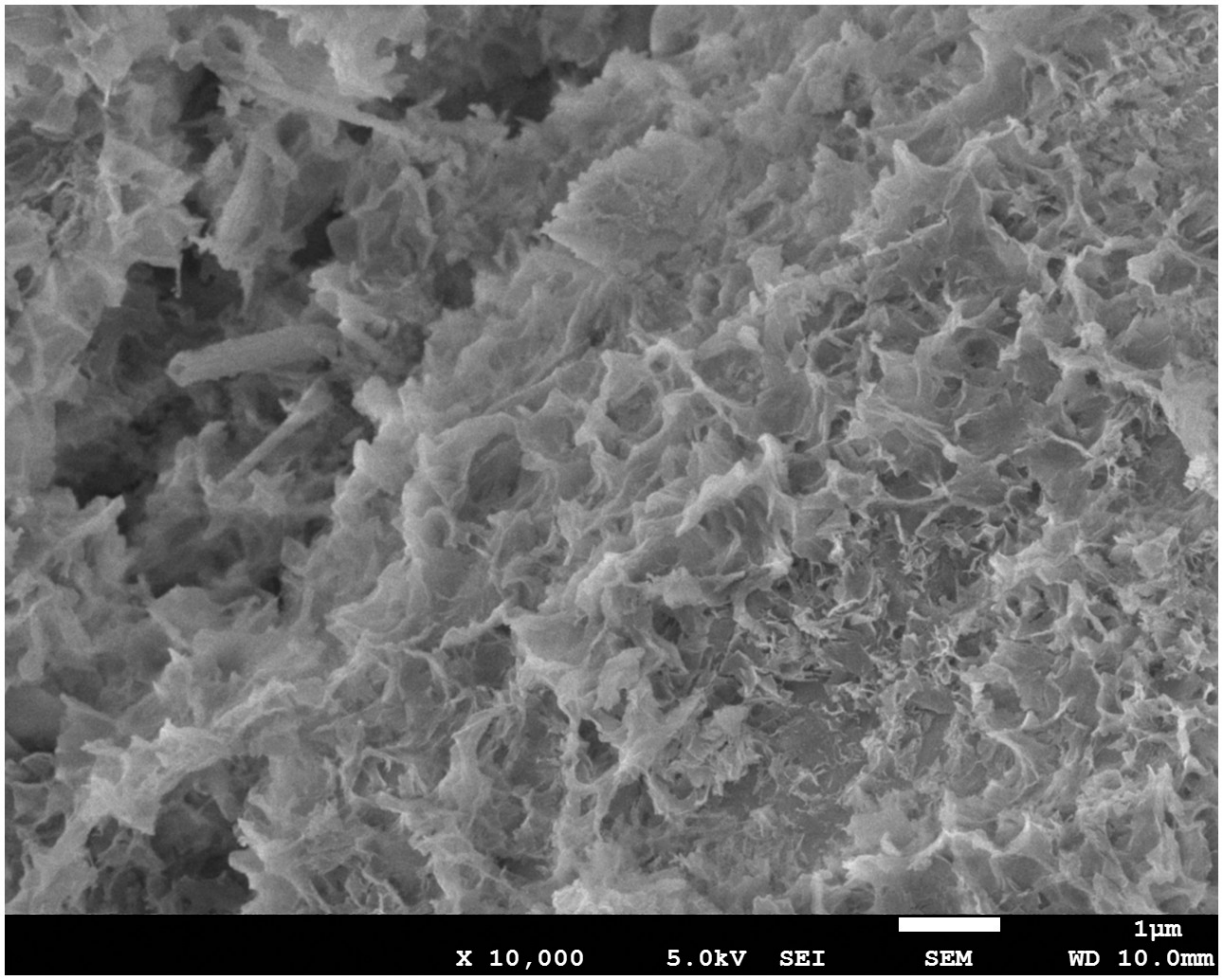
C-S-H gel (Calcium silicate hydrate).

1. The dates indicate that a series of physical and chemical changes in the backfill body, especially the hydration reaction, mainly occurred in the first 28 days. In this process, fine particles such as fly ash and cement formed a cementitious structure with a flocculent network (Fig 2), which enclosed and consolidated the gangue particles together, thus showing an obvious strength characteristic.
2. The X-ray analysis of the gangue-based cemented backfill body show a large amount of high-strength minerals such as quartz, kaolinite, illite and so on [41]. These high-strength minerals enclosed by C-S-H gel form a skeletal structure that support gangue-based cementitious body shows strength significantly.

## Analysis

**Fig 3:**
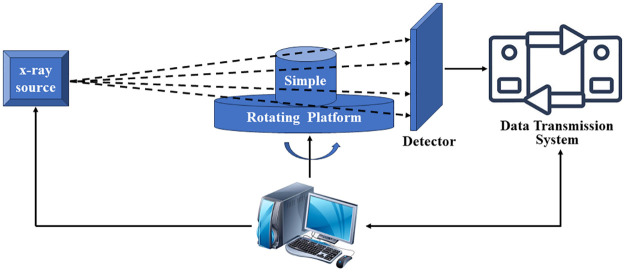
CT system.

CT scanning can present the internal state of damaged cementitious backfill body without contact. Thus, the reasons of cementitious backfill body breakdown could be research microscopically and exactly. The mechanism of CT scanning is shown as Fig 3.

### Pore and crack properties of backfill body

**Fig 4:**
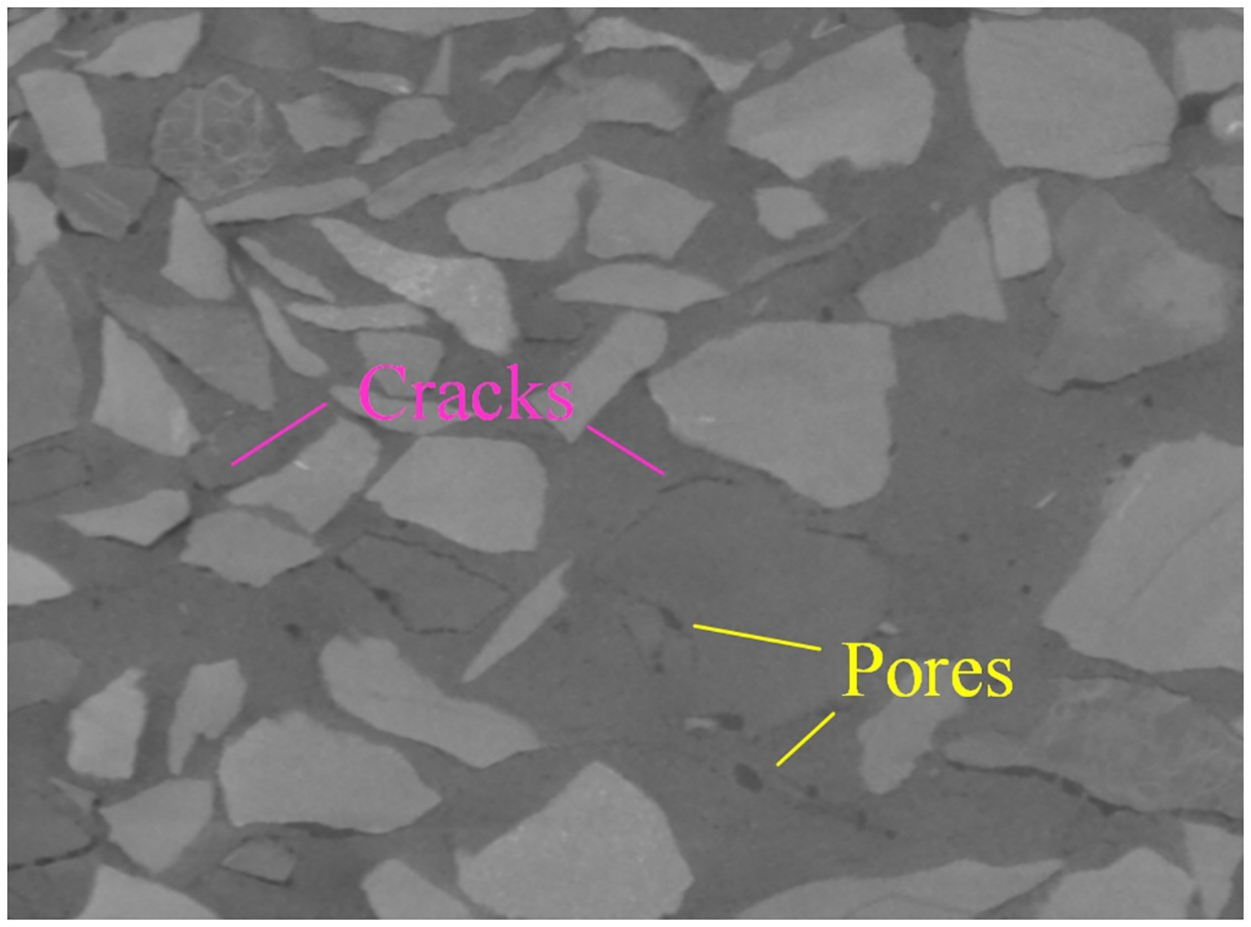
Pores and cracks in samples.

The surface of the samples on day 28 was relatively rough and full of pores of different sizes and width cracks [41]. The same phenomenon also existed inside the samples, which can be demonstrated by CT scanning results, as shown in Fig 4. MIP analysis of the samples showed that there was a large number of 0~50μM diameters. The pore diameters conformed to the normal distribution, and the maximum proportion of pores with a diameter of 20*μM* [41].

### CT scanning results

The parameter settings of the CT scanner are shown in Table 4.

**Table 4:** CT scanning parameters.

Type	Parameter settings
Scanning voltage/KV	430
Scanning electric/mA	1.55
Maximum working power/kW	0.70
Scanning spacing/mm	0.50
Enlargement factor	2.02
Scanning mode	Linear array scanning

#### Same-particle-sizes sample versus different loads

**Fig 5:**
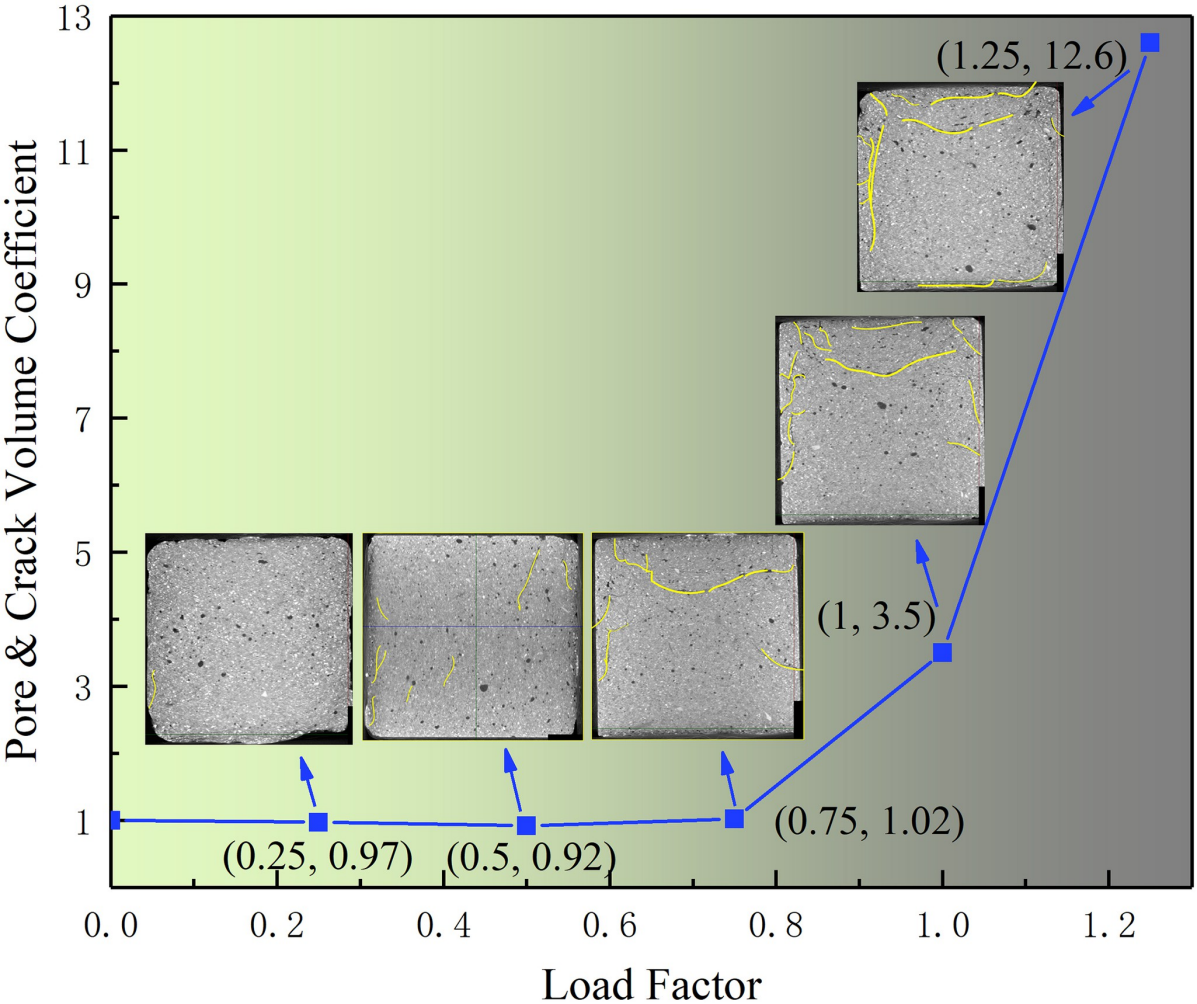
Curve of pore & crack volume versus load.

**Fig 6:**
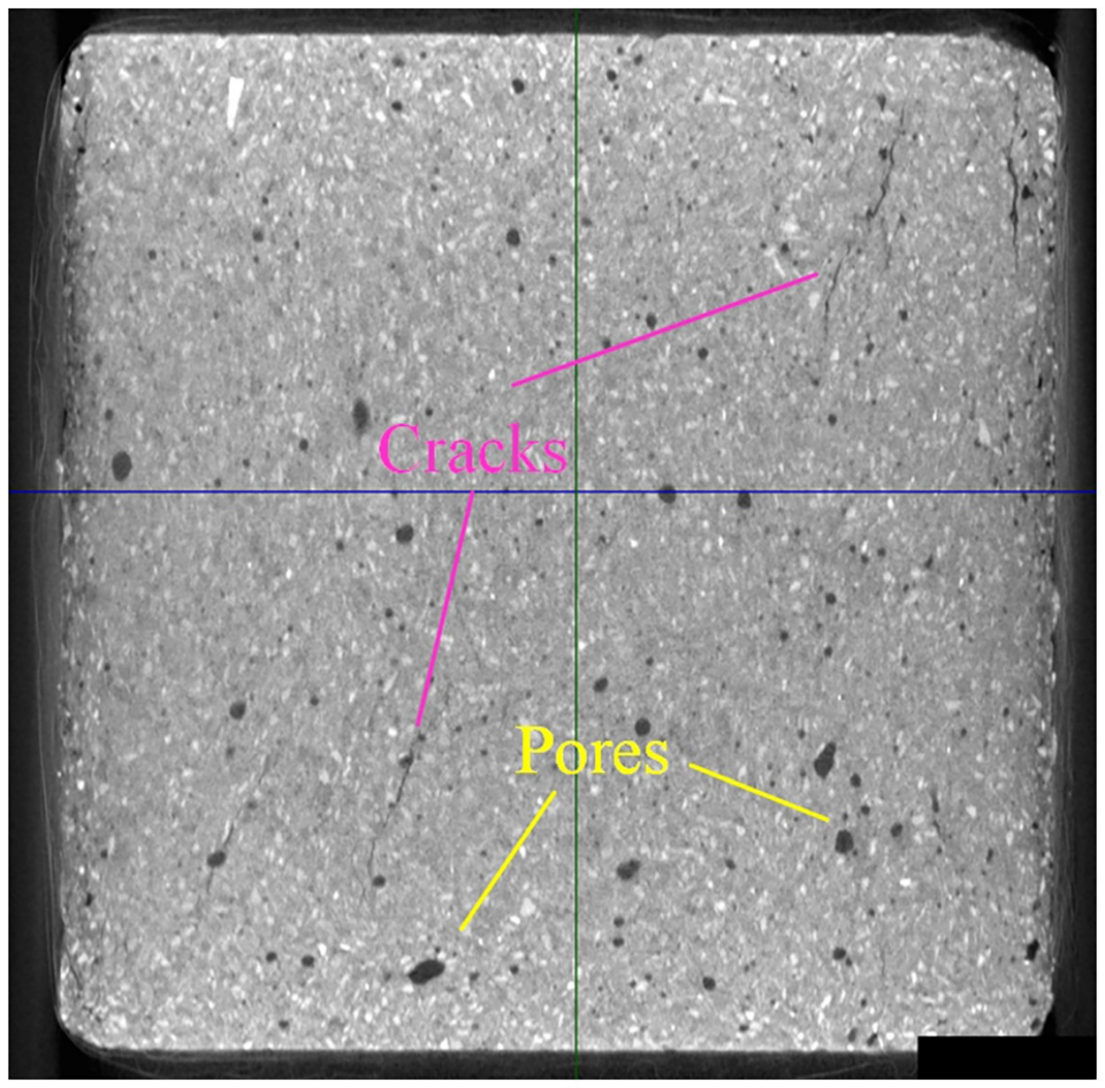
Distribution of internal pores and cracks.

**Fig 7:**
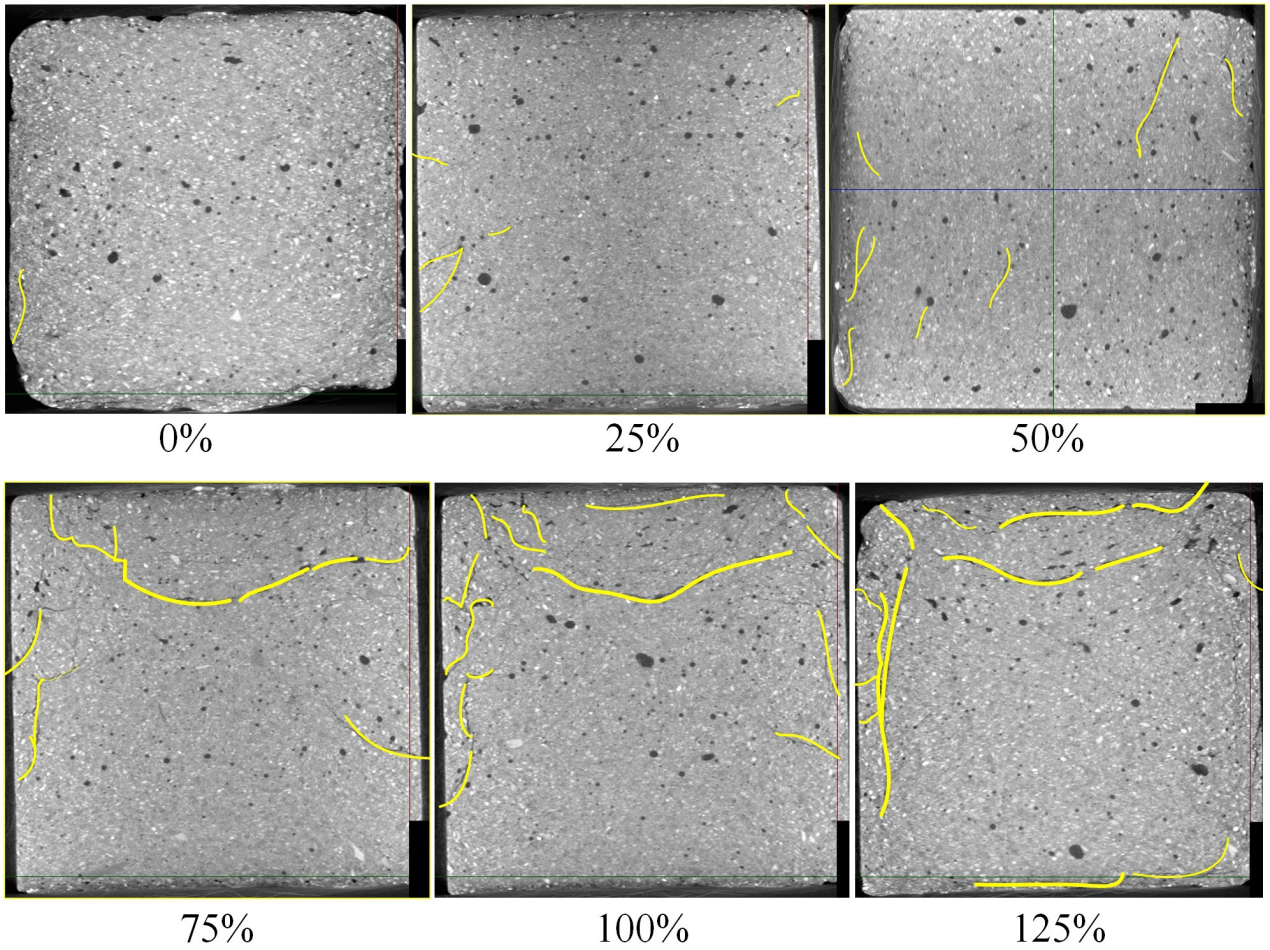
Pores & cracks.

**Fig 8:**
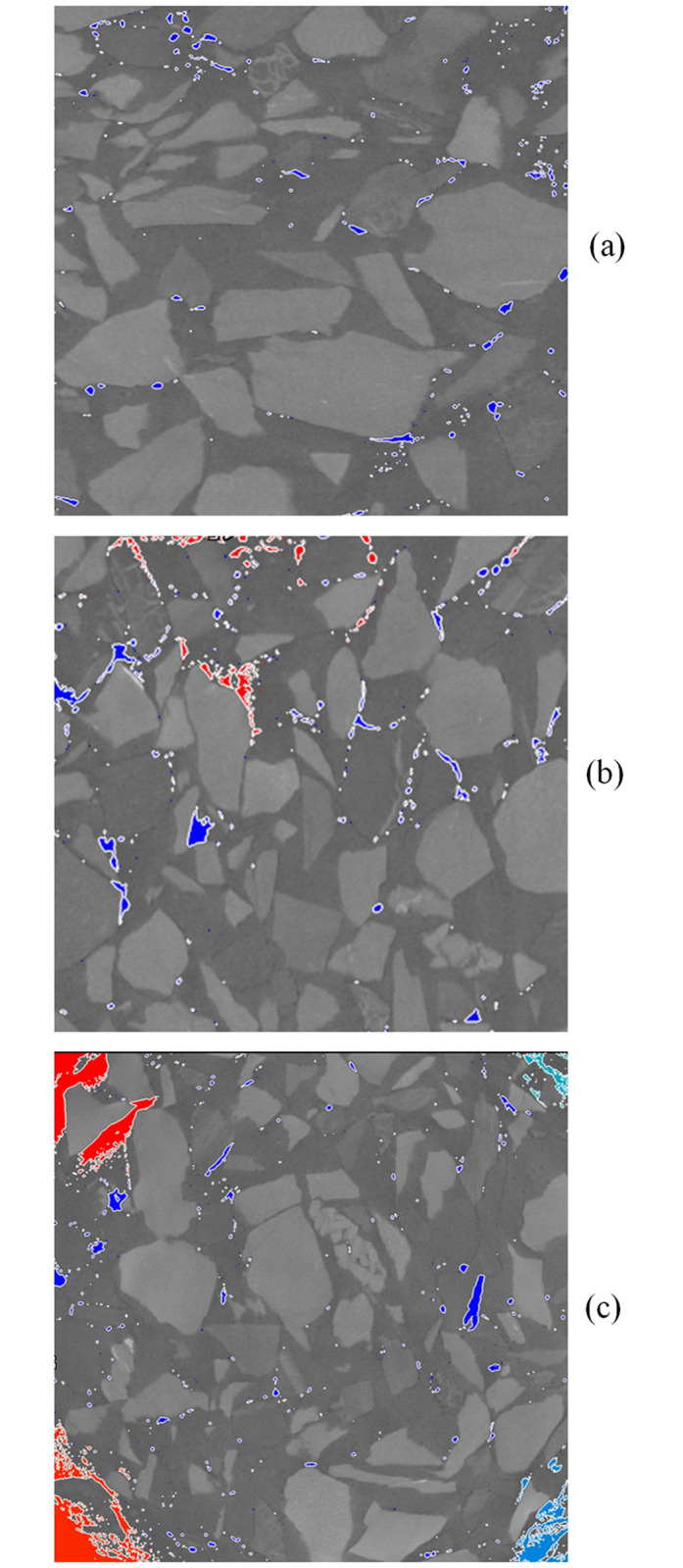
Pore & cracks versus loads of the same particle size.

**Fig 9:**
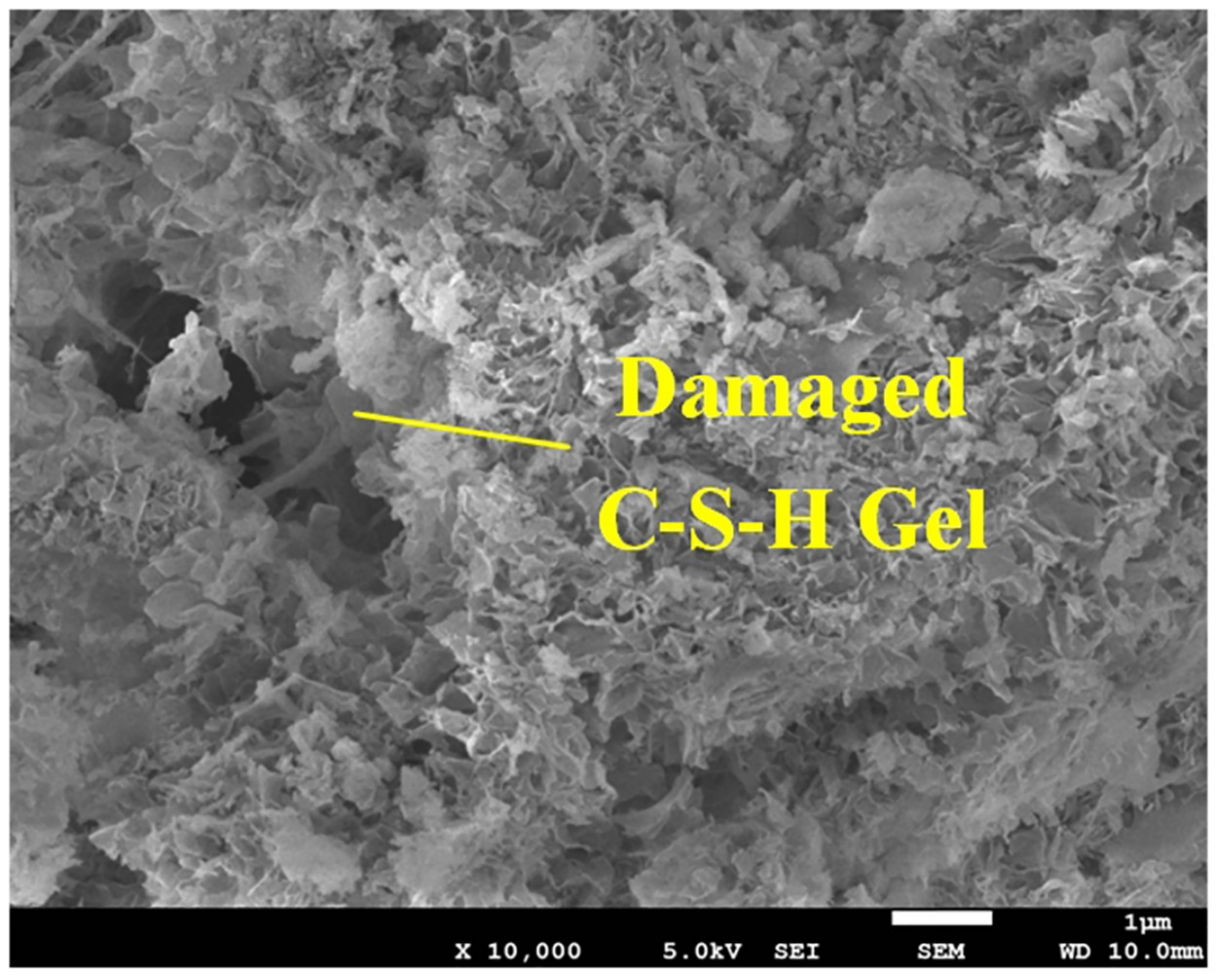
Damage C-S-H gel.

Taking E group samples as example, scan the samples which had conducted under loads of 0%, 25%, 50%, 75%, 100%, and 125% peak strength by CT. In the results, different material densities exhibited different colors. As the density of gangue was about 2200kg/m^3^ and the density of fine-particle-consolidated body was about 1800kg/m^3^, they exhibited indistinct color differences. The CT scan results for the pore and crack distribution inside the samples versus the different loads are shown as Fig 5. The curve shows the volume changes in the pore and cracks of the samples under different loads. The coordinates marked on the curve represent the load at that point and the corresponding pore and crack volume values. Fig 6 shows the distribution of internal pores and cracks versus loads. Yellow lines are used for display the length and width of cracks more clearly. The changes in the pores and cracks under different loads show the influence of load on the internal structure of the sample. The sample scanning results under different fracturing conditions show the following:

1. The distribution of cracks inside the backfill body had overall uniformity and local non-uniformity. Specifically, overall uniformity refers to the uniform distribution of large and small cracks within backfill body, and local non-uniformity refers to the significantly higher degree of crack development in an interface structure area formed by gangue particles compared with other areas. As shown in Fig 7, the cracks were mainly on the surface of the gangue, and adjacent cracks may connect and become larger (as shown by the red area in the figure).
2. The Fig 8 shows the variations in pores and cracks versus the load. When the load was less than 50% of the peak strength, the sample was in the elastic deformation stage, without yielding or failure, and the internal pores were in a compressed state. Meanwhile, the development of cracks was not significant. As the blue area (pores) became smaller or even disappeared. When the load exceeded 50% of the peak strength, the pores of the samples were compressed and tended to stabilize, and crack development was more obvious. In particular, after the load reached and exceeded the peak strength, the size of cracks increased rapidly, showing a significant positive correlation with the increase in the external load. This is shown in the figure below, as the red area (cracks) increased rapidly.
3. As shown in Figs 7 and 8, the more cracks developed, the worse the strength of the sample. The curve shows a significant negative correlation between the crack volume and strength; that is, the larger the crack volume, the lower the strength. The maximum size of the same crack significantly developed, and the total size of the crack also significantly increased. As the load increased, the length and width of the crack increased significantly. The non-connected cracks developed into a semi-network and a network. The results indicate that the main reason for the failure of the sample manifested as an increase in the scale of the internal cracks.
4. SEM (scanning electron microscopy) figure shows that the main manifestation of crack development at the interface structure was the damage C-S-H gel at that location. As Fig 9 shows. The figure shows that the C-S-H gel structure was "torn". The damaged C-S-H gel structure appeared as cracks at the macrolevel. The higher the density of cracks, the more damaged the C-S-H gel structures appeared; the greater the width of the cracks, the more thoroughly the C-S-H gel structure was damaged.
5. The cracks were mainly concentrated at the edge of the sample, which may be related to unidirectional axial compression.

#### Different particle sizes samples versus same loads

In order to highlight the differences in the morphology and scale of the internal cracks under the same load, all samples were conducted load experiments with peak strength of 125%. The purpose was to display the differences in the morphology and scale of the internal cracks under the same load. This research will highlight the impact of the interface structure formed by gangue particles on the development of cracks in samples.

1. In the elastic deformation stage, as the load increased, the surface cracks extended inward, and the length and width also increased accordingly.
2. In a larger deformation stage, under the same load, the larger the particle size of the gangue in the sample, the faster the development of cracks, and the length and width of cracks are also relatively large. This indicates that the interface structure has a significant impact on the development of cracks.
3. When the interface structure is confined, the formed cracks easily interconnect, leading to rapid expansion in the crack size.
4. All of the cracks exhibited a similar change trend.

## Conclusion

The paper revealed the relationship between morphology of pores and cracks versus load by CT scan figures.

1. When the load below peak intensity, volume of micropore decreases due to compression, but the cracks change is not significant. When the load reaches peak intensity, the cracks extended from the surface of the samples to the interior, the length and width of the cracks increased significantly, and connect as a network. That means the load had a significant impact on the morphology of micropores and cracks inside the sample.
2. There was a weak correlation between the failure of the sample and the micropores, but obvious correlation with the development of cracks. More developed and larger of the cracks, more obvious the failure characteristics of the samples. In particular, when the cracks connected as a network, the strength decreased significantly and vice versa.
3. The interface structure formed by coal gangue has an obvious positive impact on the development and scale of cracks. In the process of sample failure, the large interface structure will quickly form cracks, while the small interface structure will form cracks slightly slower. These cracks will intersect and connect with other cracks to form a network of cracks. Based on these phenomena, the particle size of gangue will affect the strength of the cementitious body at a certain extent.
4. The relationship between gangue particles and the strength of the samples indicates the influence of the structure face formed by gangue particles on the strength of the test block. Taking group C particle size as the boundary point, the strength of small particle size sample keeps an approximately stable trend with the particle size increase, the strength of large particle size samples decreases with the particle size increase. On the premise of meeting the requirements of strength and economy, the particle size of gangue should be as small as possible.
5. There is a "bonding structure" between the C-S-H structure and the gangue particles near the interface structure, which is also an important reason for the strength formation of the backfill body. Meanwhile, low strength of "bonding structure" is the initial cause of strength failure. The study mentioned that the size of the structural face (essentially the size of the "bonding structure") has a significant effect on the strength of the sample. The strength evaluation and indicator weights of C-S-H, "bonding structure", and the skeleton structure formed by the gangue particles wrapped by C-S-H need to be studied in the future. That may require a large number of experiments to explorer.

## Supporting information

S1 File (RAR)
